# Incidence and mortality rates of colorectal cancer in Malaysia

**DOI:** 10.4178/epih.e2016007

**Published:** 2016-03-09

**Authors:** Muhammad Radzi Abu Hassan, Ibtisam Ismail, Mohd Azri Mohd Suan, Faizah Ahmad, Wan Khamizar Wan Khazim, Zabedah Othman, Rosaida Mat Said, Wei Leong Tan, Siti Rahmah @ Noor Syahireen Mohammed, Shahrul Aiman Soelar, Nik Raihan Nik Mustapha

**Affiliations:** 1 Department of Medicine, Sultanah Bahiyah Hospital, Alor Setar, Malaysia; 2 Clinical Research Centre, Sultanah Bahiyah Hospital, Alor Setar, Malaysia; 3 Department of Surgery, Sultanah Bahiyah Hospital, Alor Setar, Malaysia; 4 Department of Radiotherapy and Oncology, Hospital Kuala Lumpur, Kuala Lumpur, Malaysia; 5 Department of Medicine, Hospital Ampang, Ampang, Malaysia; 6 Department of Pathology, Sultanah Bahiyah Hospital, Alor Setar, Malaysia

**Keywords:** Colorectal neoplasms, Incidence, Mortality, Ethnic groups, Sex, Malaysia

## Abstract

**OBJECTIVES:**

This is the first study that estimates the incidence and mortality rate for colorectal cancer (CRC) patients in Malaysia by sex and ethnicity.

**METHODS:**

The 4,501 patients were selected from National Cancer Patient Registry-Colorectal Cancer data. Patient survival status was cross-checked with the National Registration Department. The age-standardised rate (ASR) was calculated as the proportion of CRC cases (incidence) and deaths (mortality) from 2008 to 2013, weighted by the age structure of the population, as determined by the Department of Statistics Malaysia and the World Health Organization world standard population distribution.

**RESULTS:**

The overall incidence rate for CRC was 21.32 cases per 100,000. Those of Chinese ethnicity had the highest CRC incidence (27.35), followed by the Malay (18.95), and Indian (17.55) ethnicities. The ASR incidence rate of CRC was 1.33 times higher among males than females (24.16 and 18.14 per 100,000, respectively). The 2011 (44.7%) CRC deaths were recorded. The overall ASR of mortality was 9.79 cases, with 11.85 among the Chinese, followed by 9.56 among the Malays and 7.08 among the Indians. The ASR of mortality was 1.42 times higher among males (11.46) than females (8.05).

**CONCLUSIONS:**

CRC incidence and mortality is higher in males than females. Individuals of Chinese ethnicity have the highest incidence of CRC, followed by the Malay and Indian ethnicities. The same trends were observed for the age-standardised mortality rate.

## INTRODUCTION

Colorectal cancer (CRC) has become the third most common cancer globally [1] and has been considered as one of the leading causes of death, particularly in Western countries [2]. In 2014, 136,830 individuals were projected to be newly diagnosed with CRC, with a mortality projection of 50,310 in the US alone [3]. Asian countries are not excluded from the rise in CRC during the past 20 years [4] and the disease has become a major health concern. In Malaysia, the National Cancer Registry Report of 2007 found CRC to be the second most common cancer [5].

Studies in East Asians in Japan, Hong Kong and Singapore have investigated the incidence of CRC. The incidence of CRC was found to be increasing among Japanese people, particularly in males [6] and an increase in the incidence of CRC was also observed in Hong Kong from 1983 until 2006 [7]. Ethnic background has been postulated to influence the risk of CRC. For example, CRC incidence and mortality rates in the US were found to be higher among Black Americans (66.90 per 100,000) compared to White Americans, Asians, Hispanics, and Indians [8].

Malaysia is a country with diverse ethnic groups and cultural systems with a population of more than 28 million, including three major groups [9]. According to Article 160, of the Federal Constitution of Malaysia, “Malay” is defined as an individual who professes the religion of Islam, habitually speaks the Malay language and conforms to Malay custom [10]. “Chinese” is defined as an individual of Chinese origin, who in Malaysia is comprised of Hokkien, Kantonis, Hakka, Hainan, Foochiew, Kwongsai, Teochew, Henghua, Hokchia, and other Chinese. Meanwhile, “Indian” in Malaysian context is defined as an individual whom descendants were originally India Muslim/Malabari, Malayali, Punjabi, Sikh, Sinhala, Tamil India, Tamil Sri Lanka, Telugu, and other Indians [9]. These three major ethnicities in Malaysia are the focus of our study. To our knowledge, this is the first study in Malaysia that estimates the incidence and mortality rate for CRC patients by sex and ethnicity.

## MATERIALS AND METHODS

This study utilised secondary data from the National Cancer Patient Registry-Colorectal Cancer (NCPR-CC). The NCPR-CC is a national clinical database that aims to systematically collect data on important aspects of CRC relevant to its prevention and treatment to enable healthcare planning, implementation and evaluation in a defined population in Malaysia. Source data providers (SDPs) represent different regions of Malaysia, i.e., the peninsular (north, central, south, and east coasts), and East Malaysia (Sabah and Sarawak). In our study, there were 34 participating centres including at least one representative hospital from each of the 14 states of Malaysia. The SDPs mainly comprised major public referral hospitals treating CRC. The Clinical Research Centre at Hospital Sultanah Bahiyah is the coordinating centre that manages the registry. All CRC cases diagnosed from January 2008 until December 2013 were included.

All data are stored in a secure database with restricted access to authorised persons only. For the purpose of this study, the database of the registry was locked on April 30, 2014. This study utilised data from 34 participating centres that were entered until April 30, 2014. All cases were histologically verified as CRC by pathology reports before being accepted to enter into the database. Initially, 7,287 cases were captured. Following data exclusion (duplicated entries, as well as cases of anal cancer or metastatic lesions to the colorectum), a total of 4,501 patients were included in this study. Patient survival status was confirmed through medical records, as well as by checking with the National Registration Department, Ministry of Home Affairs. This study was registered with National Medical Research Registry and approved by Medical Research Ethical Committee (NMRR-07-49-242).

Data analyses were performed using IBM SPSS version 20.0 (IBM Corp., Armonk, NY, USA). Descriptive statistics were generated to present the frequency and percentage of CRC incidence and mortality by age group, sex, ethnicity, primary cancer site, and patient survival status. Cross tabulation was used to get the number of age category among sex and ethnic by patient status. Age-standardised rates (ASRs) were calculated from the results of cross tabulation in order to compare cancer incidence and mortality with adjusted age-specific rates within different sex and ethnicities in Malaysia, as well as to perform international comparisons.

The ASR is defined as the rate of CRC cases (incidence) and death (mortality) per 100,000 in a population with a standard age structure. The ASR was calculated by using the direct approach method as described by the following equation:

$$ASR\;=\sum r_{i}\left(\frac{n_{is}}{\sum n_{is}}\right)$$

where *r_i_* is the weight from the World Health Organization world standard population in the *i*th age class [11] and *n_is_* is the weight from the *i*th age class of the population from the Department of Statistics, Malaysia [9].

## RESULTS

There were 4,501 cases of CRC reported in the NCPR-CC from 2008 to 2013. Table 1 shows that among these cases, 55.9% were male and 42.9% were female. In terms of ethnicity, the majority were Malay (42.7%), followed by Chinese (40.3%), and Indian (5.8%).

Table 2 shows that the age-standardised incidence rate of CRC per 100,000 was highest among Chinese (27.35), compared to Malay and Indian (18.95 and 17.55 per 100,000, respectively). The age-standardised incidence rate of CRC was about 1.33 times higher among male than female (24.16 and 18.14 per 100,000, respectively). There were 2011 (44.7%) deaths recorded among the 4,501 CRC cases during the six-year period (Table 1). The mortality rate per 100,000 was highest among Chinese (11.85), followed by Malay (9.56), and Indian (7.08). The age-standardised mortality rate of CRC was 1.42 times higher among males compared to females (11.46 and 8.05 per 100,000, respectively).

## DISCUSSION

As Malaysia is a multiethnic country, it is our interest to study the incidence and mortality rate of CRC among its three main ethnicities, i.e., Malay, Chinese, and Indian. The overall age-standardised incidence rate for CRC in Malaysia was highest amongst those of Chinese ethnicity. A similar finding was reported from the population-based Singapore cancer registry (2008 to 2012), such that the age-standardised incidence rate for CRC was higher in people with Chinese ethnicity compared to Malays and Indians [12]. As the Singaporean Chinese were of the same origin as the Malaysian Chinese who migrated from China more than three generations ago, the observation on ethnic differences suggests that genetic factors may play a major role in the aetiology of CRC [12].

Our study also found that the age-standardised incidence of CRC is higher in males than females. Despite geographical variation, similar findings have been observed worldwide. The highest estimated difference in the rate of CRC was observed in Australia/New Zealand (44.8 and 32.2 per 100,000 in males and females, respectively), and a similar difference was observed in Western Africa (4.5 and 3.8 per 100,000 in males and females, respectively) [1]. In Malaysia, the same trend was observed in hepatocellular cancer, which is the second most common cancer after CRC in Malaysia and is 3.40 times likely to occur in males than females [5,13]. The reason why females exhibit a lower risk for CRC is unclear, but several studies have suggested that females hormones may have a protective effect against CRC by way of changes in bile synthesis and secretion that lead to a reduced concentration of bile acids in the colon [14,15].

The mortality rate mirrors the CRC incidence in that those of Chinese ethnicity had the highest CRC incidence and mortality rate. Meanwhile, the age-standardised mortality rate in males was 1.42 times higher than females (11.46 and 8.05 per 100,000, respectively). Global cancer statistics indicate that developed nations have an age-standardised mortality rate of 15.1 per 100,000, while in South East Asia, the age-standardised mortality rate for CRC is 15.2 per 100,000 for males and 12.9 per 100,000 for females [16].

The GLOBOCAN project report demonstrated that the overall incidence of CRC in Malaysia was the third highest (18.30 per 100,000) in South East Asia. For comparison, rates in neighbouring countries are as follows: Singapore (33.70 per 100,000), Brunei (25.00 per 100,000), the Philippines (13.10 per 100,000), Indonesia (12.80 per 100,000), and Thailand (12.40 per 100,000). Brunei exhibited the highest mortality rate (12.0 per 100,000) followed by Singapore, Malaysia, Indonesia, the Philippines, and Thailand (11.8, 9.4, 8.6, 7.8, and 7.3 per 100,000, respectively) [1]. Among these nations, survival is greatest in Singapore, Brunei and Malaysia with a mortality-to-incidence rate of 0.35, 0.48, and 0.51, respectively. Meanwhile, survival is generally poor for Indonesia, the Philippines, and Thailand.

According to Parkin, it is important for a registry database to review their data completeness index, as only a high degree of completeness will ensure that reported figures such as incidence and survival rate are close to their true value [17,18]. It was suggested that one possible way to report the overall data completeness of a registry is by calculating the mortality per incidence (M:I) ratio. We demonstrated the index of completeness by assessing the M:I ratio in our dataset, where we compared the number of deaths, which were obtained from a source independent to the registry (the National Registration Department, Ministry of Home Affairs), against the number of cases reported for the period of time being studied (2008 to 2013). Shin et al. [19] reported that the M:I ratio for CRC in males and females was 0.37 and 0.43, respectively. Our overall CRC M:I ratio was found to be 0.47 and 0.44 for males and females, respectively, which verified the completeness of the data in our registry.

To our knowledge, this study is the most representative data of the incidence and mortality of CRC in different sex and ethnicities in Malaysia. In our study, out of 34 centres that contributed to the data, there was at least one representative hospital from each 14 states of Malaysia where most of the centres are the major referral public hospitals treating CRC. We have reported the overall incidence of CRC in Malaysia and demonstrated that it was higher in Chinese than in Malay and Indian ethnicities. Similarly, the Chinese ethnicity showed the highest CRC mortality rate. Incidence and mortality rates were both higher in males.

As disease prevention is always better than cure, it is very important for policy makers to consider CRC prevention in Malaysia in depth. For instance, early interventions such as health education and implementation of a targeted CRC screening programme should be considered, as it has been postulated to decrease the rate of CRC incidence in several countries. It is our hope that these findings will provide insight to policy makers in devising effective strategies to improve CRC disease control in Malaysia.

## Figures and Tables

**Table 1.** Descriptive statistics of colorectal cancer cases in the National Cancer Patient Registry-Colorectal Cancer, Malaysia, 2008 to 2013

Variable	n (%)
Age (yr)	
<19	11 (0.2)
20-24	17 (0.4)
25-29	47 (1.0)
30-34	65 (1.4)
35-39	112 (2.5)
40-44	194 (4.3)
45-49	328 (7.3)
50-54	465 (10.3)
55-59	627 (13.9)
60-64	713 (15.8)
65-69	650 (14.4)
70-74	616 (13.7)
>75	613 (13.6)
Not reported	43 (1.0)
Sex	
Male	2,518 (55.9)
Female	1,933 (42.9)
Not reported	50 (1.1)
Ethnicity	
Malay	1,922 (42.7)
Chinese	1,814 (40.3)
Indian	259 (5.8)
Other	484 (10.8)
Not reported	22 (0.5)
Primary cancer site	
Left-sided	3,480 (77.3)
Right-sided	770 (17.1)
Not reported	251 (5.6)
Patient status	
Survived	2,490 (55.3)
Died	2,011 (44.7)

**Table 2.** Colorectal cancer incidence and mortality of cases in the National Cancer Patient Registry-Colorectal Cancer, 2008 to 2013, by sex and ethnicity

Characteristic	Incidence (/100,000)			Mortality (/100,000)			Mortality/Incidence ratio		
	Overall	Male	Female	Overall	Male	Female	Overall	Male	Female
Overall	21.32	24.16	18.14	9.79	11.46	8.05	0.46	0.47	0.44
Ethnicity									
Malay	18.95	21.79	16.09	9.56	11.56	7.57	0.50	0.53	0.47
Chinese	27.35	30.77	23.22	11.85	13.47	10.07	0.43	0.44	0.43
Indian	17.55	21.43	13.71	7.08	9.63	4.78	0.40	0.45	0.35
